# Breast ductal carcinoma *in situ* presenting as recurrent non-puerperal mastitis: case report and literature review

**DOI:** 10.1186/1477-7819-11-179

**Published:** 2013-08-07

**Authors:** Yee Vonne Liong, Ga Sze Hong, Jennifer Gek Choo Teo, Geok Hoon Lim

**Affiliations:** 1 Breast Department, KK Women’s and Children’s Hospital, Singapore, Singapore; 2 Department of Pathology, KK Women’s and Children’s Hospital, Singapore, Singapore

**Keywords:** Breast ductal carcinoma *in situ*, Non-puerperal mastitis, Recurrent mastitis

## Abstract

Breast ductal carcinoma *in situ* (DCIS) is a preinvasive form of breast cancer. It typically presents as microcalcifications which are picked up on screening mammogram. We report an atypical case of breast DCIS presenting with recurrent non-puerperal mastitis with a normal mammogram and perform a literature review.

## Background

Breast ductal carcinoma *in situ* (DCIS) is typically asymptomatic and presents as abnormal microcalcifications picked up on screening mammogram. In this study, we report an unusual presentation of DCIS as recurrent non-puerperal mastitis with a normal mammogram. Though the underlying causal relationship between breast cancer and mastitis in this case remains unclear, this case report highlights the importance of having a high level of clinical suspicion in these cases of atypical presentation so that breast cancer will not be missed.

## Case presentation

A 61-year-old postmenopausal woman presented with right outer breast pain, redness and swelling of 3 days duration. Mammogram was essentially normal at that time. Ultrasound of her right breast revealed no masses but increased echogenicity and skin thickening in the outer half of her breast, likely due to inflammation. Clinical impression was that of right mastitis. She was treated with 1 week of oral antibiotics and responded with resolution of her symptoms. A follow-up ultrasound performed 1 month later showed improvement of the inflammatory changes in the right breast.

The patient then remained well until she presented 8 months later with another similar episode of right mastitis over the previously affected area. An ultrasound performed this time again showed skin thickening in the right outer breast with underlying inflammatory changes. No discrete nodule or collection was seen. The patient’s symptoms again improved with antibiotics with complete resolution of the redness and swelling of her right breast.

**Figure 1:**
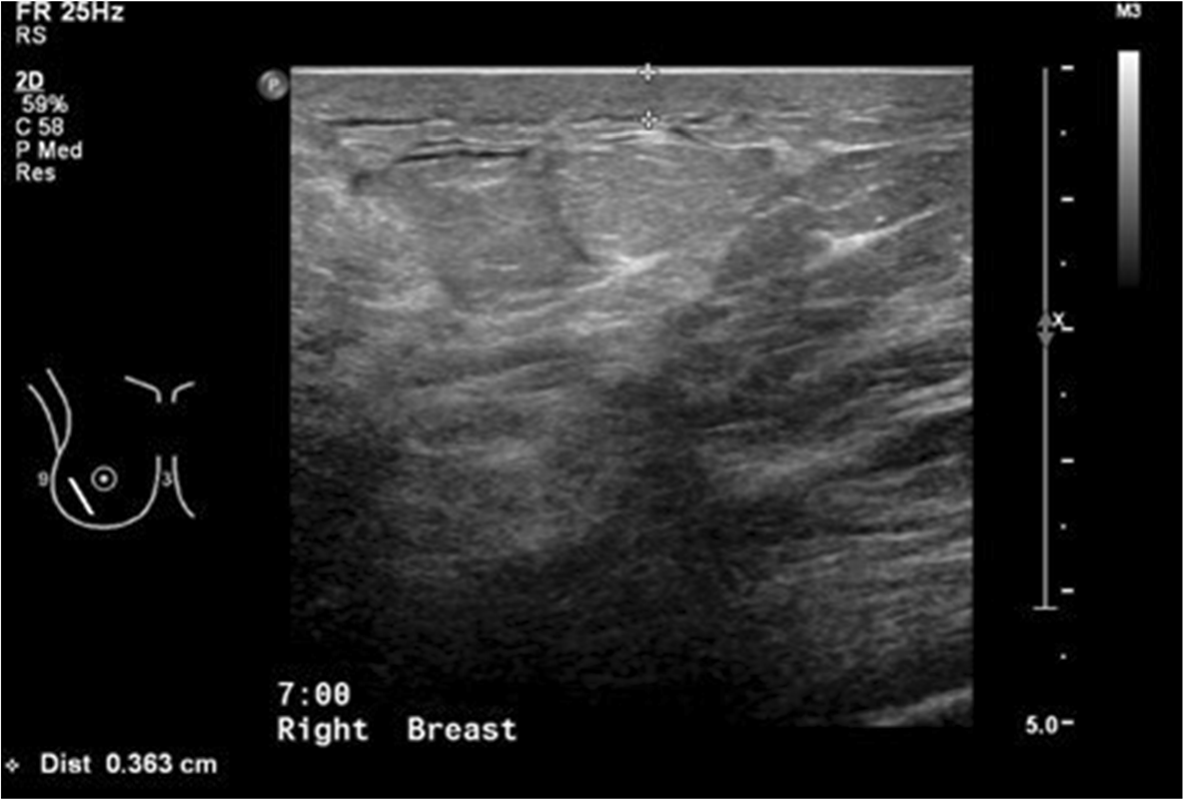
Right breast ultrasound showing skin thickening and underlying marked inflammatory changes with increased echogenicity.

However, the patient returned 6 months later presenting with another similar episode of right breast redness, swelling and pain. Physical examination then revealed right nipple retraction and signs of inflammation over the previous area of interest. Repeat ultrasound (Figure 1) showed marked inflammatory changes in the right breast with skin thickening, suggestive of an inflammatory process. Though the patient’s symptoms again responded with antibiotics, an underlying breast malignancy, especially inflammatory breast cancer, needed to be excluded in view of recent nipple retraction.

A skin punch biopsy and core biopsy of the inflamed breast tissue were carried out which revealed mild superficial dermatitis on skin biopsy. Core biopsy showed DCIS with focal chronic mastitis.

**Figure 2:**
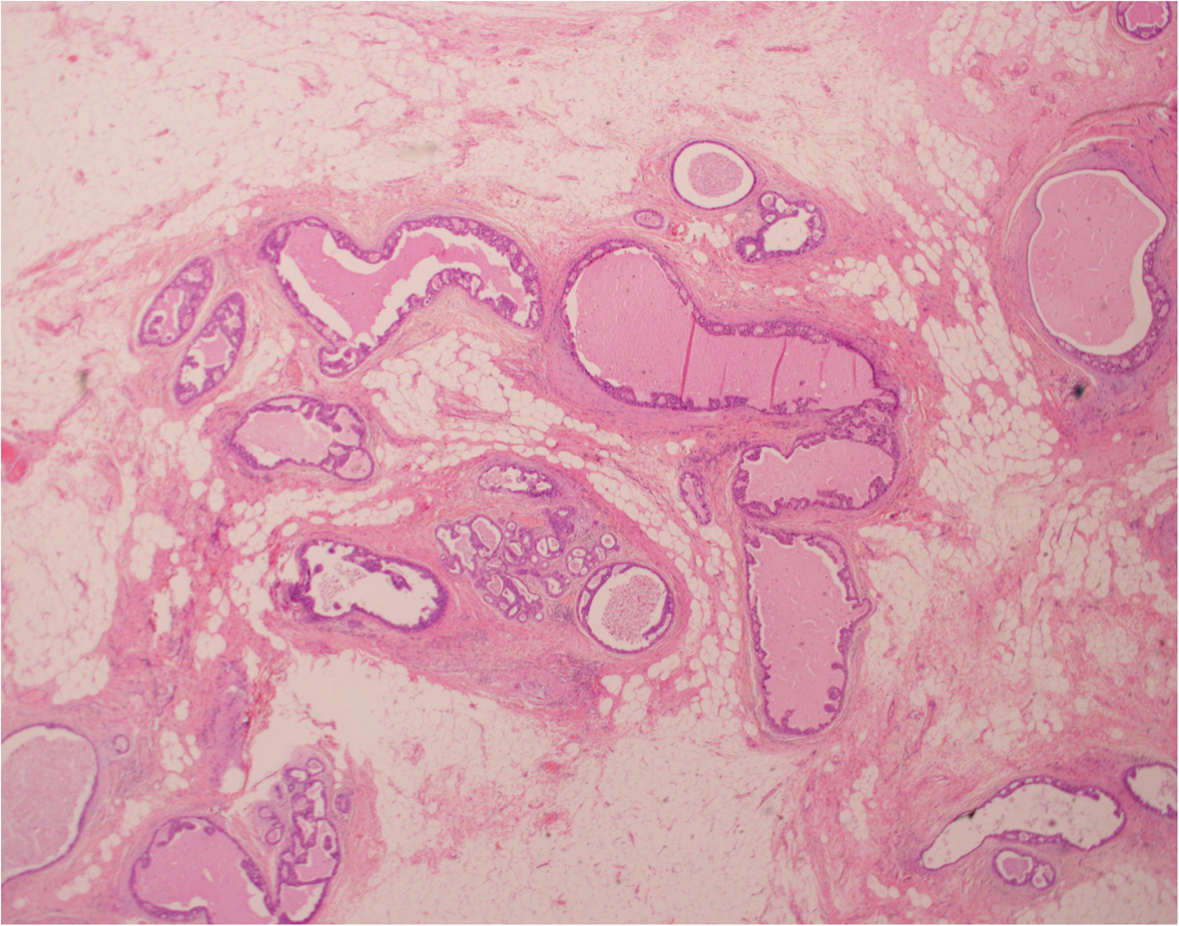
**Hematoxylin and eosin stain, original magnification ×20.** Ducts with high-grade ductal carcinoma *in situ*, some showing cystic hypersecretory features.

The patient underwent a right total mastectomy and sentinel lymph node biopsy. Final histology showed a 75 mm high-grade DCIS with areas of necrosis. There was, however, no invasive component. Some of the involved ducts (up to 50%) showed features of cystic hypersecretory DCIS, characterized by cystically dilated ducts containing thyroid colloid-like eosinophilic secretions (Figure 2). There was no lymph node involvement and the margins were clear. The estrogen and progesterone receptors status were both negative. Her2 neu score was positive. Her postoperative recovery was uneventful and the patient did not require any further treatment.

## Discussion

DCIS, a precursor of invasive ductal carcinoma, consists of proliferation of cancerous cells within the lumens of the breast duct with no invasion beyond the epithelial basement membrane [1].

Before the widespread use of screening mammogram, DCIS often presented with a palpable breast mass or thickening or nipple discharge or diagnosis of Paget’s disease of the nipple [2]. Currently, however, DCIS tends to be clinically asymptomatic and is picked up in nearly 90% of cases on mammograms as microcalcification (76%), soft tissue densities (11%) or both (13%) [3].

The presentation of non-puerperal mastitis as non-inflammatory breast cancer or DCIS is rare. The true incidence of these cases is unknown, though it was demonstrated that up to 1.81% of women with non-puerperal mastitis could eventually develop non-inflammatory breast cancer 1 year following their mastitis [4]. In this study, however, the site of non-inflammatory breast cancer is at a location distant to the infection lesion, unlike our patient in the case report. Peters F. et al., therefore suggests that non-puerperal mastitis may be a risk factor for breast cancer, though the underlying mechanism is unclear [4]. Another cohort study also showed an overall slightly increased risk of breast cancer in women with a history of mastitis but also could not find a causal relationship between inflammation and the development of breast cancer [5]. It remains unclear if non-inflammatory breast cancer or DCIS was the cause of mastitis or *vice versa*.

The pathophysiology of DCIS manifesting as mastitis is unclear. One possible explanation suggested by Damiani S et al. is that high grade DCIS has been associated with damage of the myoepithelial cell layer and basement membrane surrounding the ductal lumen [6]. This resultant nidus of dead ductal tissue then acted as a source for chronic infection [7], hence resulting in the atypical manifestation of DCIS as mastitis.

In our case, the tumor had characteristerics of cystic hypersecretory DCIS, a rare histological variant of intraductal cancer [8]. This lesion typically shows a low-grade behavior but can potentially become invasive and metastasize. It does not, however, have any characteristic clinical or mammographic features or prognostic significance. Our atypical presentation of DCIS as mastitis may be related to recurrent infection of the necrotic areas within the DCIS.

An important differential diagnosis for consideration in our case was inflammatory breast cancer which is often misdiagnosed as mastitis. It is rare and accounts for approximately 2.5% of all breast cancer cases [9]. Radiologically, it can sometimes present with very subtle skin thickening without an overt mass. In fact, skin thickening has been reported to be the most common radiographic finding on mammogram and ultrasound for inflammatory breast cancer [10]. Hence, the diagnosis of inflammatory breast cancer is often made based on the clinical picture and pathognomonic feature of tumor emboli blocking the dermal lymphatic channels on histology [9]. In our case, the skin punch and core biopsy excluded inflammatory breast cancer.

Though ultrasound features of extensive skin thickening and infiltrated malignant nodes and mammographic features of diffuse skin thickening and increased density could help in differentiation of malignant mastitis over the non-infectious or simple mastitis [11], non-puerperal mastitis can often have no specific mammographic or sonographic signs [12] which are diagnostic. As a result, a biopsy is still needed for a diagnosis and to exclude malignant mastitis if clinically warranted.

## Conclusion

DCIS can present atypically as recurrent mastitis without significant suspicious imaging features. A pathological investigation such as a biopsy may be necessary to exclude breast malignancy in these rare cases, especially if there is recurrent presentation of non-puerperal mastitis and other associated sinister clinical features.

## Consent

Informed consent was obtained from the patient for the publication of this case report and any accompanying images.

## Abbreviations

DCIS: Ductal carcinoma *in situ.*

## Competing interests

The authors declare that they have no competing interests.

## Authors’ contributions

YVL prepared the preliminary draft of this case report. GSH conceived of the study and edited the manuscript. JGCT provided the pathology figure and edited the pathology component of the paper. GHL drafted and revised the manuscript. All authors read and approved the final manuscript.
